# Impact of Vancomycin Treatment and Gut Microbiota on Bile Acid Metabolism and the Development of Non-Alcoholic Steatohepatitis in Mice

**DOI:** 10.3390/ijms24044050

**Published:** 2023-02-17

**Authors:** Kaichi Kasai, Naoya Igarashi, Yuki Tada, Koudai Kani, Shun Takano, Tsutomu Yanagibashi, Fumitake Usui-Kawanishi, Shiho Fujisaka, Shiro Watanabe, Mayuko Ichimura-Shimizu, Kiyoshi Takatsu, Kazuyuki Tobe, Koichi Tsuneyama, Yukihiro Furusawa, Yoshinori Nagai

**Affiliations:** 1 Department of Pharmaceutical Engineering, Faculty of Engineering, Toyama Prefectural University, 5180 Kurokawa, Imizu 939-0398, Japan; 2 Toyama Prefectural Institute for Pharmaceutical Research, 17-1 Nakataikouyama, Imizu 939-0363, Japan; 3 First Department of Internal Medicine, University of Toyama, 2630 Sugitani, Toyama 930-0194, Japan; 4 Institute of Natural Medicine, University of Toyama, 2630 Sugitani, Toyama 930-0194, Japan; 5 Department of Pathology and Laboratory Medicine, Tokushima University Graduate School of Biomedical Sciences, 3-8-15 Kuramoto-cho, Tokushima 770-8503, Japan

**Keywords:** antibiotic, bile acid, cholic acid, fibrosis, gut microbiota, inflammation, macrophage, metronidazole, non-alcoholic steatohepatitis, vancomycin

## Abstract

The potential roles of the gut microbiota in the pathogenesis of non-alcoholic fatty liver disease, including non-alcoholic steatohepatitis (NASH), have attracted increased interest. We have investigated the links between gut microbiota and NASH development in Tsumura-Suzuki non-obese mice fed a high-fat/cholesterol/cholate-based (iHFC) diet that exhibit advanced liver fibrosis using antibiotic treatments. The administration of vancomycin, which targets Gram-positive organisms, exacerbated the progression of liver damage, steatohepatitis, and fibrosis in iHFC-fed mice, but not in mice fed a normal diet. F4/80^+^-recruited macrophages were more abundant in the liver of vancomycin-treated iHFC-fed mice. The infiltration of CD11c^+^-recruited macrophages into the liver, forming hepatic crown-like structures, was enhanced by vancomycin treatment. The co-localization of this macrophage subset with collagen was greatly augmented in the liver of vancomycin-treated iHFC-fed mice. These changes were rarely seen with the administration of metronidazole, which targets anaerobic organisms, in iHFC-fed mice. Finally, the vancomycin treatment dramatically modulated the level and composition of bile acid in iHFC-fed mice. Thus, our data demonstrate that changes in inflammation and fibrosis in the liver by the iHFC diet can be modified by antibiotic-induced changes in gut microbiota and shed light on their roles in the pathogenesis of advanced liver fibrosis.

## 1. Introduction

Non-alcoholic fatty liver disease (NAFLD) is characterized by the hepatic manifestation of metabolic disorders. NAFLD encompasses a wide range of liver pathologies, from non-alcoholic fatty liver (NAFL) to non-alcoholic steatohepatitis (NASH) and cirrhosis [1]. The etiology of NAFLD involves both genetic predisposition and epigenetic regulation by environmental factors [1]. The multiple parallel-hit hypothesis, which states that multiple factors play a role in its pathogenesis, has been widely accepted [2]. Besides genetic and environmental factors, the internal environment and various metabolites, such as gut microbiota and bile acid (BA), require further investigation to fully elucidate the pathogenesis of NAFLD [3].

BAs are steroid-based molecules that are synthesized from cholesterol in the liver. They are involved in nutritional lipid absorption and function as signaling molecules to regulate the metabolism via BA-sensing receptors, including the nuclear farnesoid X receptor (FXR) and cell membrane Takeda G protein-coupled receptor 5 (TGR5) (also known as GPBAR1 (G protein-coupled membrane receptor 1)) [4,5,6]. Gut microbiota can contribute to secondary BA synthesis and BA metabolism by structural modulation, including deconjugation, dehydroxylation, oxidation, and desulfation [7]. In the large intestine, microbes modify hydroxyl groups on the steroid ring and produce various secondary BAs [8]. The level and composition of BA play roles in intestinal barrier function [9,10]. The increased expression levels of hepatic BA synthesis genes in rats on a high-fat diet (HFD) elevate 12α-hydroxylated (12αOH) BA concentrations, such as cholic acid (CA), in enterohepatic circulation [11,12]. The microbial *baiB* gene is involved in 7α-dehydroxylation in the large intestine, which produces deoxycholic acid (DCA), a secondary 12αOH BA [13]. Furthermore, high DCA levels induced leaky gut in mice on an HFD [14]. Of note, the liver is continuously exposed to BAs via the enterohepatic circulation, and this exposure is associated with liver disease, including liver cancer [15].

Many studies have focused on identifying the specific roles of gut microbiota in metabolic disorders. Gut dysbiosis is closely associated with a decrease in beneficial short-chain fatty acid-producing bacteria, changes in the composition of BAs [16], and activation of immune responses against lipopolysaccharides (LPS) [17]. Changes in BA and inflammatory signaling, insulin resistance, and glucose metabolism induced by an HFD were shown to be modified by antibiotic-induced changes in gut microbiota [18]. Changes in gut microbiota that affect the gut–liver axis are also associated with the progression of chronic liver disease, advanced fibrosis, and hepatocellular carcinoma (HCC) [19,20,21,22,23,24]. For example, gut sterilization and germ-free conditions can decrease the formation of HCC in mouse models [15,25]. In humans, *Ruminococcaceae* and *Veillonellaceae* are the main microbial taxa associated with significant liver fibrosis in non-obese subjects [26]. In the *Ruminococcaceae*, *Ruminococcus faecis* alleviated fibrosis in mouse NAFLD models [26], and *Faecalibacterium prausnitzii*, also in the *Ruminococcaceae*, was reported to regulate the hepatic fat content and composition of lipid species and reduce adipose tissue inflammation in HFD-fed mice [27].

Histologically, NASH is characterized by steatosis, lobular inflammation, hepatocellular ballooning, and fibrosis of the liver. In humans, fibrosis in NASH typically first appears in zone 3 as a “chicken-wire” pattern that spreads to the portal area, ultimately leading to portal–portal and portal–central bridging fibrosis [28]. However, currently used animal NASH models, such as the methionine- and choline-deficient diet models and carbon tetrachloride (CCl4)-induced model, do not reflect human dietary habits and are not characterized by histological changes similar to those of human NASH, such as bridging fibrosis. A high-fat/cholesterol/cholate-based (iHFC) diet that is not deficient in specific nutrients induced advanced fibrosis that spreads to the portal area of Tsumura-Suzuki non-obese (TSNO) mice [29]. Furthermore, iHFC-fed TSNO mice exhibited stage 3 bridging fibrosis [29]. Therefore, iHFC diet-induced NASH in TSNO mice may be a more representative model of human NASH than current animal models. In our previous study, we also characterized the dynamics of hepatic macrophage subsets in iHFC-fed TSNO mice and investigated their roles in the development of advanced liver fibrosis [30]. The fluorescence-activated cell sorter analysis showed that the recruited macrophages consisted of two distinct subsets: CD11c^+^/Ly6C^−^ and CD11c^−^/Ly6C^+^ cells [30]. Furthermore, the histological and RNA sequence analyses indicated that CD11c^+^/Ly6C^−^ cells promoted liver fibrosis and hepatic stellate cell activation, whereas CD11c^−^/Ly6C^+^ cells played roles in anti-inflammation and tissue repair [30].

The aim of this study was to dissect the links between gut microbiota, liver inflammation, and fibrosis using two antibiotics that target different organisms. We found that vancomycin, which targets Gram-positive organisms, exacerbated the progression of liver damage, steatohepatitis, and fibrosis in iHFC-fed TSNO mice, but not in mice fed a normal diet. F4/80^+^-recruited macrophages were more abundant in the liver of vancomycin-treated mice. The infiltration of CD11c^+^-recruited macrophages into the liver, forming hepatic crown-like structures (hCLSs), was enhanced by vancomycin treatment. The co-localization of this macrophage subset with collagen in the liver of iHFC-fed TSNO mice was greatly augmented in vancomycin-treated mice. These changes were rarely seen in iHFC-fed TSNO mice with metronidazole, which targets anaerobic organisms. Finally, vancomycin treatment dramatically modulated the level and balance of BA composition in the iHFC-fed TSNO mice, by decreasing bile salt hydrolase (BSH) and major bacterial BA dehydroxylases, which are mainly produced by Gram-positive bacteria. We concluded that the effects of vancomycin on the exacerbation of NASH development depend on an interaction of the gut microbiome, BA metabolism, and inflammatory responses in the liver of the iHFC-fed TSNO mice.

## 2. Results

### 2.1. Modification of Gut Microbiota by Vancomycin Exacerbates iHFC Diet-Induced Liver Damage and Lipid Metabolism in TSNO Mice

To investigate the relationship between gut microbiota and the development of NASH, we challenged TSNO mice with an iHFC diet, and modified the microbiome by treatment with either metronidazole (MTZ) or vancomycin (VCM) (Figure S1). MTZ is an absorbable antibiotic that targets anaerobic organisms, whereas VCM is a non-absorbable antibiotic that targets primarily Gram-positive organisms. These antibiotics are frequently used to treat *Clostridium difficile* infection and are commonly used in patients with inflammatory bowel disease. A 16S rRNA sequence analysis of fecal samples was performed to determine the bacterial composition of TSNO mice after 4 weeks on an iHFC diet, with either placebo or antibiotic treatment. The α-diversity and principal component analyses showed clear differences in the microbial communities between mice fed a normal diet (ND) and iHFC-fed mice with placebo (iHFC + P) or between iHFC + P and iHFC-fed mice with antibiotic treatment (iHFC + MTZ and iHFC + VCM) (Figure S2A,B). Despite initial differences in microbiota among these four groups, the gut microbiota of mice in both the ND and iHFC + P groups were dominated by *Firmicutes*, whereas in the iHFC + MTZ group, *Bacteroidetes* accounted for 57% of the bacterial sequences (Figure S2C). In the iHFC + VCM group, *Bacteroidetes* were reduced to 0%, and this reduction was associated with an increased abundance of *Proteobacteria* and *Deferribacteres* (Figure S2C). A heatmap of the relative abundance of 16S sequences shows that VCM treatment killed most Gram-positive bacteria, such as *Ruminococcus* and *Lachnospiraceae*, short-chain fatty acid-producing bacteria (Figure S3). Conversely, Gram-negative bacteria such as *Parasutterella*, which are involved in BA metabolism, and *Escherichia*, typical LPS-possessing bacteria, increased and became dominant by VCM treatment (Figure S3). *Clostridium* are Gram-positive bacteria that may have survived VCM treatment because they formed spores or were highly resistant (Figure S3).

**Figure 1:**
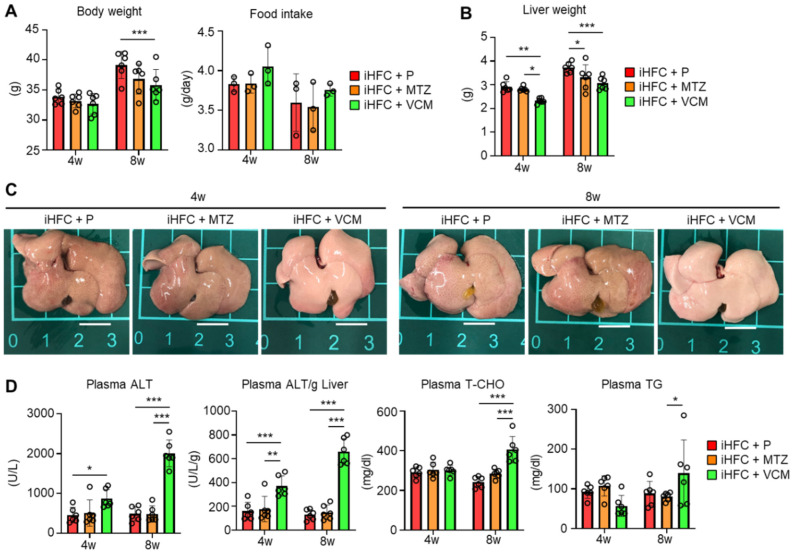
VCM treatment exacerbates liver damage in iHFC diet-fed Tsumura-Suzuki non-obese (TSNO) mice. (**A**) Body weights (n = 6 per group) and daily food intakes (n = 3 per group) were measured for placebo (iHFC + P)-, MTZ (iHFC + MTZ)-, or VCM (iHFC + VCM)-treated TSNO mice fed the iHFC diet. (**B**) Liver weights of placebo- or antibiotic-treated TSNO mice fed the iHFC diet (n = 6 per group). (**C**) Representative photos of the livers from placebo- or antibiotic-treated TSNO mice fed the iHFC diet. Scale bars, 1 cm. (**D**) Plasma ALT, T-CHO, and TG levels were measured (n = 6 per group). Plasma ALT levels per liver weight (g) were also calculated (n = 6 per group). Data are shown as means ± SD. * *p* < 0.05, ** *p* < 0.01, *** *p* < 0.001.

We also examined whether antibiotic treatment affected liver weight and liver damage in iHFC-fed mice. The body mass was significantly decreased by VCM treatment only at 8 weeks of feeding (Figure 1A, left), and no significant differences were found in the average daily food intake between the placebo and antibiotic groups (Figure 1A, right). For liver weight, there was a significant difference between the placebo and antibiotic groups or between the MTZ and VCM groups (Figure 1B). The liver weight of VCM-treated mice was significant decreased at both 4 and 8 weeks of treatment (Figure 1B). The liver from iHFC-fed mice was pale, as reported previously (Figure 1C) [30], and here we found that the VCM-treated mice had much paler livers than the placebo- and MTZ-treated mice (Figure 1C). There were marked increases in the plasma alanine aminotransferase (ALT) activity of the iHFC-fed mice after 4 and 8 weeks of VCM treatment (Figure 1D). The iHFC + VCM mice also had high plasma total cholesterol (T-CHO) and triglyceride (TG) concentrations compared with mice in the other groups (Figure 1D).

To determine whether the effects of VCM treatment on the liver were due to antibiotic-induced liver damage, we treated ND-fed mice with placebo or antibiotics. MTZ and VCM affected only the body weight of these mice; no other parameter was affected (Figure S4). Thus, VCM treatment exacerbated liver damage and lipid metabolism in iHFC-fed TSNO mice.

### 2.2. Modification of Gut Microbiota by Vancomycin Exacerbates iHFC Diet-Induced Inflammation, Steatosis, Hepatocyte Ballooning, and Fibrosis in the Liver of TSNO Mice

**Figure 2:**
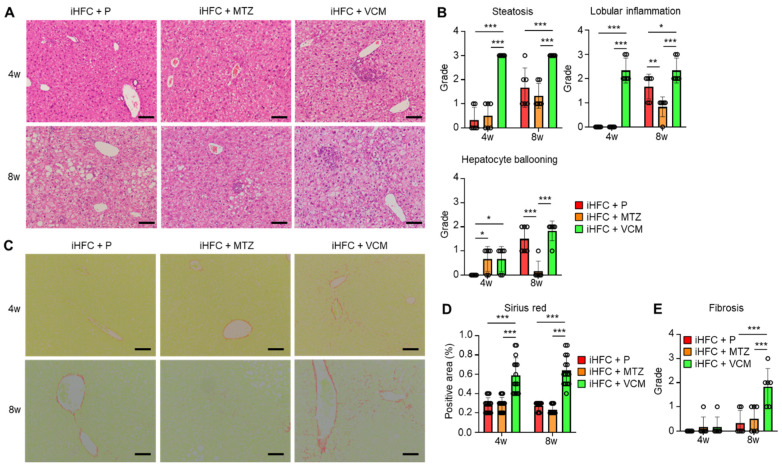
VCM treatment exacerbates iHFC diet-induced steatohepatitis and fibrosis in the liver of TSNO mice. (**A**) Representative images of hematoxylin and eosin-stained sections of the liver from placebo- or antibiotic-treated TSNO mice fed the iHFC diet. Scale bars, 100 μm. (**B**) Steatosis (0 to 3), lobular inflammation (0 to 3), and hepatocyte ballooning (0 to 2) were assessed according to the criteria proposed by Kleiner et al. as described in Materials and Methods (n = 6 per group). (**C**) Representative images of Sirius red-stained sections of the liver from placebo- or antibiotic-treated TSNO mice fed the iHFC diet. Scale bars, 100 μm. (**D**) Five locations were photographed in each of three sections in each group. Then, positive areas for Sirius red were measured at 15 locations using ImageJ software Version 1.53t, and the mean and SD were calculated. (**E**) Liver fibrosis (0 to 4) was assessed according to the criteria proposed by Kleiner et al. as described in Materials and Methods (n = 6 per group). Data are shown as means ± SD. * *p* < 0.05, ** *p* < 0.01, *** *p* < 0.001.

To further investigate these findings, we performed a histopathological analysis of the liver from mice in the iHFC + P, iHFC + MTZ, and iHFC + VCM groups. We observed mild steatosis after 4 weeks in the iHFC + P, and the steatosis worsened as the time on the iHFC diet increased (Figure 2A,B). Lobular inflammation and hepatocyte ballooning were observed from 8 weeks (Figure 2A,B). These histopathological changes were significantly exacerbated after 4 weeks of VCM treatment (Figure 2B), and lobular inflammation and hepatocyte ballooning were significantly improved after 8 weeks of MTZ treatment (Figure 2B). We also examined iHFC diet-induced fibrotic changes in the liver of placebo- or antibiotic-treated mice. The Sirius red-positive areas in the sections from VCM-treated mice were significantly higher than those of placebo- or MTZ-treated mice from 4 weeks of treatment, indicating fibrosis was most apparent after VCM treatment (Figure 2C,D). The fibrosis gradually expanded, with bridging fibrosis becoming apparent after 8 weeks of VCM treatment similar to that after 24 weeks on the iHFC diet (Figure 2C,E) [29,30]. Thus, VCM treatment exacerbated steatohepatitis and fibrosis in the liver of iHFC diet-fed TSNO mice.

### 2.3. Modification of Gut Microbiota by Vancomycin Increases the Levels of Inflammation- or Fibrosis-Related Gene Expression in the Liver of iHFC Diet-Fed TSNO Mice

**Figure 3:**
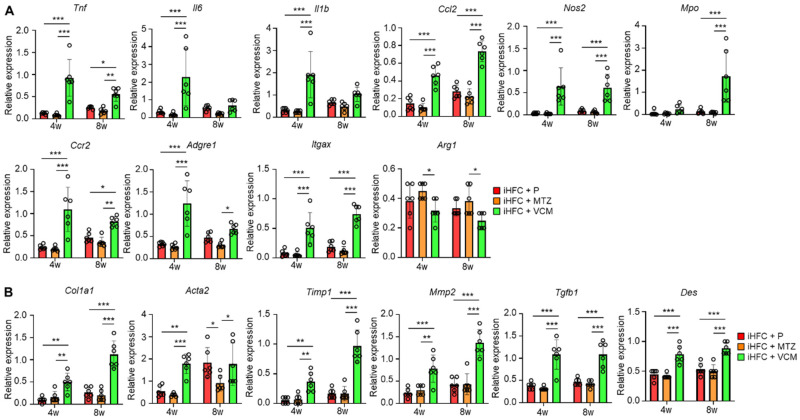
VCM treatment increases the levels of inflammation- or fibrosis-related gene expression in the liver of iHFC diet-fed TSNO mice. (**A**) RT-qPCR of TNF-α (*Tnf*), IL-6 (*Il6*), IL-1b (*Il1b*), CCL2 (*Ccl2*), iNOS (*Nos2*), MPO (*Mpo*), CCR2 (*Ccr2*), F4/80 (*Adgre1*), CD11c (*Itgax*), and Arg-1 (*Arg1*) mRNA in the livers of placebo- or antibiotic-treated TSNO mice fed the iHFC diet (n = 6 per group). (**B**) RT-qPCR of collagen type 1 (*Col1a1*), αSMA (*Acta2*), TIMP-1 (*Timp1*), MMP2 (*Mmp2*), TGF-β (*Tgfb1*), and Desmin (*Des*) mRNA in the livers of TSNO mice fed the iHFC diet (n = 6 per group). Data are shown as means ± SD. * *p* < 0.05, ** *p* < 0.01, *** *p* < 0.001.

We measured the expression levels of inflammatory and fibrotic genes in the liver of mice in the iHFC + P, iHFC + MTZ, and iHFC + VCM groups. The expression levels of pro-inflammatory genes (*Tnf*, *Il6*, and *Il1b*), chemokine and its receptor genes (*Ccl2*, (C-C motif) ligand 2, and *Ccr2*), neutrophil granulocyte marker (*Mpo*), macrophage marker (*Adgre1*), and M1 macrophage markers (*Itgax* and *Nos2*) were markedly increased by VCM treatment (Figure 3A), whereas the expression level of M2 macrophage marker (*Arg1*) was significantly decreased by VCM treatment compared with their expression by MTZ treatment (Figure 3A). Consistent with the histological data (Figure 2C,D), the expression levels of collagen 1 (*Col1a1*) and alpha-smooth muscle actin (αSMA) (*Acta2*) mRNAs were higher in the liver of VCM-treated mice than they were in the placebo- or MTZ-treated mice (Figure 3B). VCM treatment also markedly increased the mRNA expression levels of the tissue inhibitor of metalloproteinase 1 (*Timp1*) and matrix metallopeptidase 2 (*Mmp2*), which regulate extracellular matrix degradation (Figure 3B). The mRNA expression of transforming growth factor beta (*Tgfb1*), which regulates extracellular matrix production, was high in the liver of VCM-treated mice (Figure 3B), and the expression of a hepatic stellate cell marker gene (*Des*) was significantly higher in the iHFC + VCM group than it was in the iHFC + P and iHFC + MTZ groups (Figure 3B). Thus, modification of the gut microbiome by VCM treatment exacerbates inflammation and fibrosis in the liver of iHFC-fed TSNO mice.

### 2.4. Modification of Gut Microbiota by Vancomycin Augments the Infiltration of CD45^+^ Leukocytes and F4/80^+^ Macrophages in the Liver of iHFC Diet-Fed TSNO Mice

**Figure 4:**
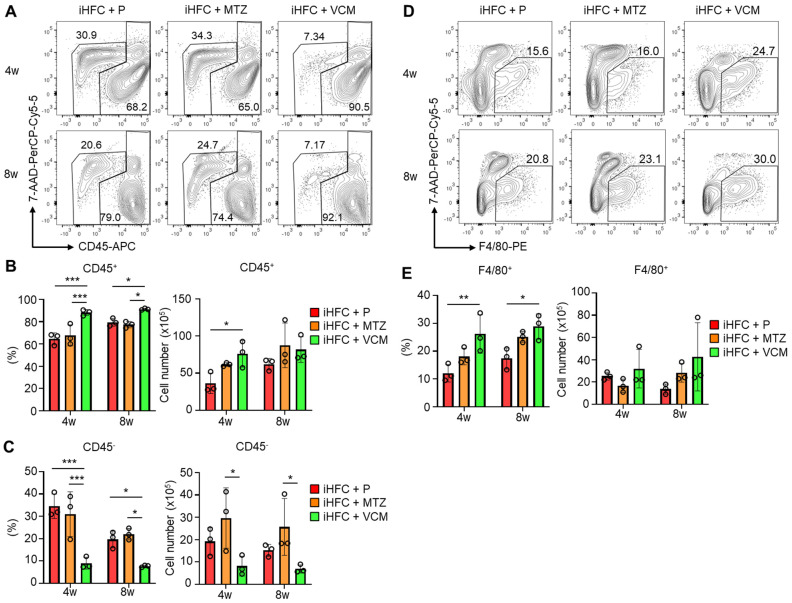
VCM treatment augments iHFC diet-induced accumulation of CD45^+^ leukocytes and F4/80^+^ macrophages in the liver of TSNO mice. (**A**) Representative flow cytometry data of CD45 expression on non-parenchymal cells including KCs and excluding 7-AAD^+^ dead cells of the liver from placebo- or antibiotic- treated TSNO mice on the iHFC diet. (**B**) Percentage (Left) and cell number (Right) of CD45^+^ live non-parenchymal cells were determined by flow cytometry analysis conducted in Figure 4A (n = 3 per group). (**C**) Percentage (Left) and cell number (Right) of CD45^−^ live non-parenchymal cells were determined by flow cytometry analysis conducted in Figure 4A (n = 3 per group). (**D**) Single non-parenchymal cells were analyzed with a plot of F4/80 and 7-AAD, and then gated on live F4/80^+^ cells excluding dead cells and highly auto-fluorescent F4/80-positive KCs. (**E**) Percentage (Left) and cell number (Right) of F4/80^+^-recruited macrophages were determined by flow cytometry analysis conducted in Figure 4D (n = 3 per group). Data are shown as means ± SD. * *p* < 0.05, ** *p* < 0.01, *** *p* < 0.001.

To investigate the roles of immune cells in the pathogenesis of the VCM-mediated exacerbation of NASH, we isolated non-parenchymal cells from the liver and counted the numbers of CD45^+^ leukocytes. No significant differences in the numbers of live non-parenchymal cells were detected between the iHFC + P and the iHFC + MTZ or iHFC + VCM group (Figure S5). A flow cytometric analysis demonstrated that the percentage of CD45^+^ cells including Kupffer cells (KCs) increased by VCM treatment compared with the percentages for the placebo or MTZ treatment (Figure 4A,B), whereas the percentage and cell number of CD45^−^ cells were lower by VCM treatment than they were for the placebo- or MTZ treatment (Figure 4A,C). We also found that the percentage of F4/80^+^ cells excluding KCs was increased by VCM treatment (Figure 4D,E). These results imply that CD45^+^ leukocytes, which include F4/80^+^ macrophages, accumulate in the liver with VCM treatment and play key roles in exacerbating inflammation and fibrosis in the liver of iHFC-fed mice.

### 2.5. Modification of Gut Microbiota by Vancomycin Augments the Infiltration of F4/80^+^-Recruited Macrophages in the Liver of iHFC-Fed TSNO Mice

**Figure 5:**
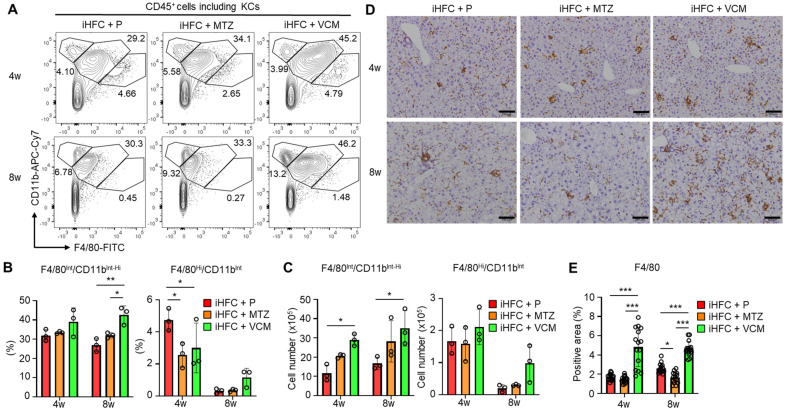
VCM treatment augments iHFC diet-induced accumulation of F4/80^+^-recruited macrophages in the liver of TSNO mice. (**A**) Representative flow cytometry data of F4/80 and CD11b expression on CD45^+^ non-parenchymal cells including KCs of the liver from placebo- or antibiotic-treated TSNO mice. (**B**) Percentage (Left) and cell number (Right) of F4/80^Hi^/CD11b^Int^ KCs were determined by flow cytometry analysis in Figure 5A (n = 3 per group). (**C**) Percentage (Left) and cell number (Right) of F4/80^Int^/CD11b^Int-Hi^-recruited macrophages were determined by flow cytometry analysis in Figure 5A (n = 3 per group). (**D**) Representative histological images of F4/80 immunostaining of the liver from placebo- or antibiotic-treated TSNO mice on the iHFC diet. Scale bars, 100 μm. (**E**) Five locations were photographed in each of three sections in each group. Then, positive areas for F4/80 were measured at 15 locations in each group using ImageJ software, and the mean and SD were calculated. Data are shown as means ± SD. * *p* < 0.05, ** *p* < 0.01, *** *p* < 0.001.

We then focused on the subsets of CD45^+^ and F4/80^+^ non-parenchymal cells in the liver of the antibiotic-treated TSNO mice. Previously, we reported that at least three populations expressing F4/80 and/or CD11b were present in ND-fed mouse liver: F4/80^−^/CD11b^Hi^ neutrophils, F4/80^Int^/CD11b^Int-Hi^-recruited macrophages, and F4/80^Hi^/CD11b^Int^ KCs [30]. We also found that the percentage of F4/80^Hi^/CD11b^Int^ KCs was reduced by feeding mice the iHFC diet [30]. The iHFC diet also induced hepatic infiltration with F4/80^Int^/CD11b^Int-Hi^ macrophages, which constitute hCLSs, suggesting that this macrophage subset might be involved in the development of hepatocyte death-induced liver fibrosis in this mouse NASH model [30]. The percentage of F4/80^Hi^/CD11b^Int^ KCs was significantly reduced by MTZ and VCM treatment after 4 weeks of treatment compared with their percentage in the placebo group (Figure 5A,B). Conversely, the percentage and cell number of F4/80^Int^/CD11b^Int-Hi^ macrophages were increased by VCM treatment (Figure 5C).

Immunohistochemical staining showed that the F4/80-positive areas of liver sections were gradually increased by iHFC-feeding [30]. In the VCM-treated mice, the F4/80-positive areas were more than those in the placebo- and MTZ-treated mice (Figure 5D,E). Interestingly, MTZ treatment significantly reduced the F4/80-positive area at 8 weeks compared with the placebo (Figure 5E). These results imply that VCM treatment augmented the infiltration of F4/80^Int^/CD11b^Int-Hi^-recruited macrophages into the liver, thereby forming hCLSs, suggesting that this macrophage subset may be involved in the VCM exacerbation of liver fibrosis in this mouse NASH model.

### 2.6. Modification of Gut Microbiota by Vancomycin Augments the Accumulation of CD11c^+^-Recruited Macrophages in the Liver of iHFC-Fed TSNO Mice

**Figure 6:**
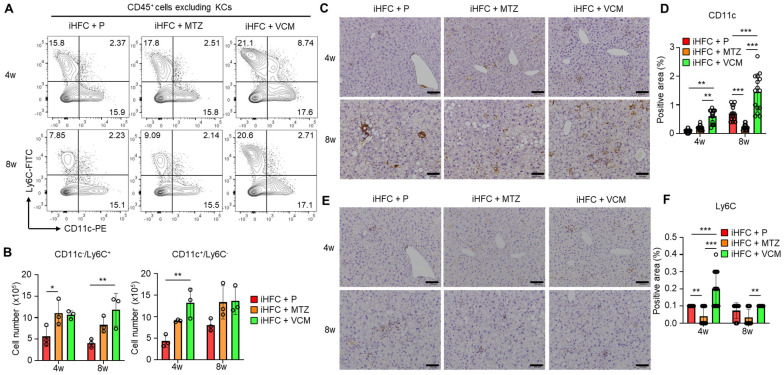
VCM treatment augments iHFC diet-induced accumulation of CD11c^+^/Ly6C^−^ and CD11c^−^/Ly6C^+^-recruited macrophages in the liver of TSNO mice. (**A**) Representative flow cytometry data of CD11c and Ly6C expression on CD45^+^ non-parenchymal cells excluding KCs of the liver from placebo- or antibiotic-treated TSNO mice. (**B**) Cell numbers of CD11c^+^/Ly6C^−^ (Left) and CD11c^−^/Ly6C^+^ (Right)-recruited macrophages were determined by flow cytometry analysis (n = 3 per group). (**C**) Representative histological images of CD11c immunostaining of the liver from placebo- or antibiotic-treated TSNO mice on the iHFC diet. Scale bars, 100 μm. (**D**) Five locations were photographed in each of three sections in each group. Then, positive areas for CD11c were measured at 15 locations in each group using ImageJ software, and the mean and SD were calculated. (**E**) Representative histological images of Ly6C immunostaining of the liver from placebo- or antibiotic-treated TSNO mice on the iHFC diet. Scale bars, 100 μm. (**F**) Five locations were photographed in each of three sections in each group. Then, positive areas for Ly6C were measured at 15 locations in each group using ImageJ software, and the mean and SD were calculated. Data are shown as means ± SD. * *p* < 0.05, ** *p* < 0.01, *** *p* < 0.001.

We previously showed that F4/80^+^-recruited macrophages in the liver of iHFC-fed mice consisted of two macrophage subsets: CD11c^+^/Ly6C^−^ and CD11c^−^/Ly6C^+^ cells [30]. The RNA sequence analysis also showed that CD11c^+^/Ly6C^−^ cells may promote liver fibrosis and hepatic stellate cell activation, whereas CD11c^−^/Ly6C^+^ cells may play an anti-inflammatory role and promote tissue repair [30]. The percentages and numbers of these two subsets were slightly increased by VCM treatment (Figure 6A). Significant increases in the cell numbers of these subsets were found in the iHFC + MTZ and iHFC + VCM groups compared with the number in the iHFC + P group (Figure 6B). An immunohistochemical analysis showed that the accumulation of CD11c^+^ and Ly6C^+^ cells when fed the iHFC diet was increased by VCM treatment (Figure 6C–F), whereas MTZ treatment significantly decreased the CD11c-positive area at 8 weeks of treatment (Figure 6D). The Ly6C-positive area was also significantly reduced by MTZ treatment at 4 and 8 weeks compared with the placebo and VCM treatment (Figure 6F).

### 2.7. Modification of Gut Microbiota by Vancomycin Augments iHFC Diet-Induced Accumulation of CD11c^+^ Cells That Are Co-Localized with Collagen Fibers in the Liver of iHFC-Fed TSNO Mice

**Figure 7:**
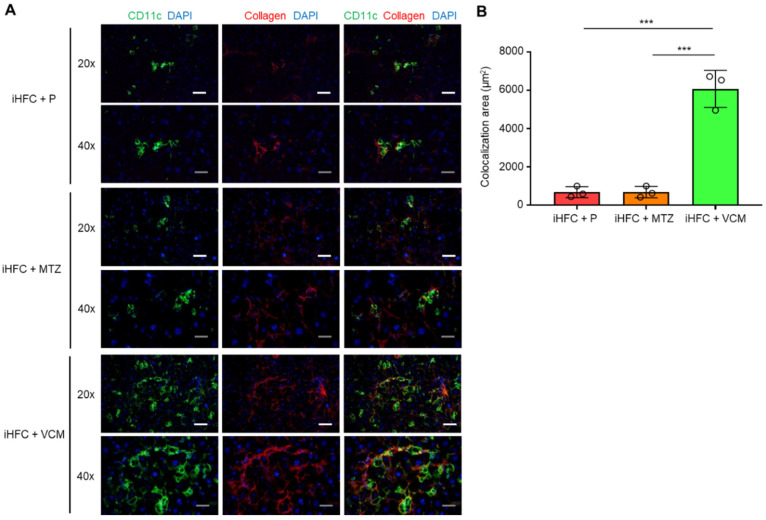
VCM treatment augments iHFC diet-induced accumulation of CD11c^+^ cells which are co-localized with collagen fibers. (**A**) Representative histological images (20× or 40× magnification) of fluorescent immunohistochemistry for CD11c, collagen type 1, and DAPI of the livers from placebo- or antibiotic-treated TSNO mice on the iHFC diet for 8 weeks. White scale bars, 100 μm. Gray scale bars, 50 µm. (**B**) Co-localization areas were calculated as described in Figure S6 and the Materials and Methods. Data are shown as means ± SD. *** *p* < 0.001.

To elucidate the role of CD11c^+^-recruited macrophages in the development of progressive liver fibrosis in VCM-treated mice, we performed immunofluorescence staining for CD11c and collagen type 1. Collagen deposition was evident in the liver at 12 weeks of feeding mice the iHFC diet [30]. Mice in the iHFC + P group had few CD11c^+^ cells and low collagen deposition at 8 weeks of feeding (Figure 7A); however, the co-localization of collagen was evident in the liver at this time point by CD11c immunostaining (Figure 7A,B). Interestingly, VCM-treated mice showed more CD11c^+^ aggregation, which forms hCLSs, and collagen deposition than the placebo-treated mice (Figure 7A), and the co-localization of collagen with CD11c in the liver was markedly enhanced by VCM treatment (Figure 7A,B). Conversely, no changes were detected between CD11c and collagen in the liver from MTZ-treated mice and placebo-treated mice by immunostaining (Figure 7A,B). These findings suggest that CD11c^+^-recruited macrophages played key roles in the development of progressive liver fibrosis in the iHFC-fed mice treated with VCM.

### 2.8. The iHFC Diet and Antibiotic Treatment Affect the Level of Fecal Bile Acid Metabolism

**Figure 8:**
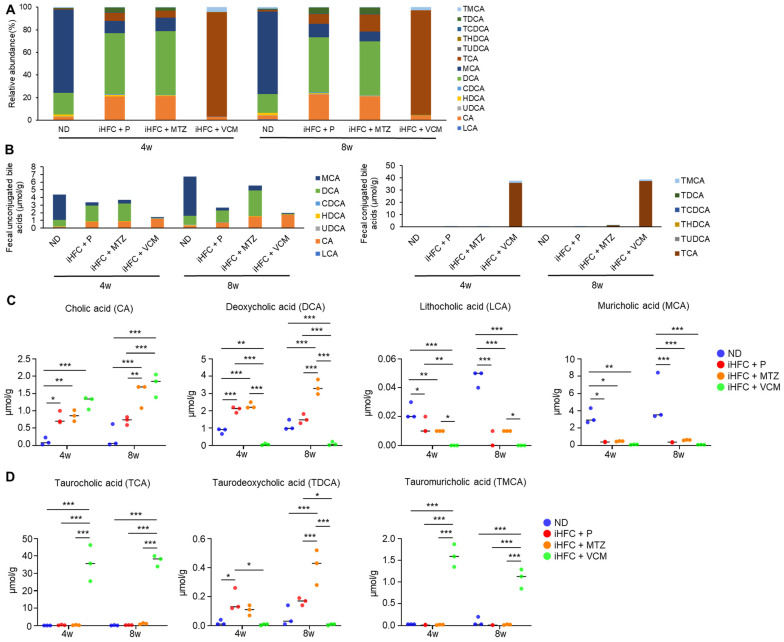
An iHFC diet and antibiotic treatment affect the levels of fecal BA metabolism. (**A**) Total BA composition in the feces of ND, iHFC + P, iHFC + MTZ, and iHFC + VCM groups after 4 and 8 weeks of treatment (n = 3 per group). (**B**) Concentration of unconjugated BAs (Left) and conjugated BAs (Right) in the feces of ND, iHFC + P, iHFC + MTZ, and iHFC + VCM groups after 4 and 8 weeks of treatment (n = 3 per group). (**C**,**D**) Changes in individual unconjugated BAs (**C**) and conjugated BAs (**D**) in the feces of ND, iHFC + P, iHFC + MTZ, and iHFC + VCM groups after 4 and 8 weeks of treatment (n = 3 per group). Data are shown as means ± SD. * *p* < 0.05, ** *p* < 0.01, *** *p* < 0.001.

To further investigate the mechanism involved in the exacerbation of liver inflammation and fibrosis in the VCM-treated mouse NASH model, we assessed the effect of the iHFC diet and antibiotic treatment on BA metabolism. We demonstrated that the iHFC diet and iHFC + VCM had major effects on the levels of fecal BA metabolites (Figure 8). Fecal BA profiles in TSNO mice revealed that an iHFC diet (iHFC + P) increased the relative abundances of cholic acid (CA) and deoxycholic acid (DCA) and decreased that of muricholic acid (MCA) (Figure 8A). Total concentrations of fecal unconjugated BAs in the iHFC + P, iHFC + MTZ, and iHFC + VCM groups were decreased compared with those in the ND group (Figure 8B, left). Notably, VCM treatment markedly reduced the total concentrations of unconjugated BAs (Figure 8B, left). Compared with the ND group, the concentration of CA, which was contained in the iHFC diet, was increased in the iHFC+ P, iHFC + MTZ, and iHFC + VCM groups, whereas the concentration of MCA was severely reduced in all three groups (Figure 8B, left). For conjugated BAs in the feces, the taurocholic acid (TCA) concentration was markedly increased in the iHFC + VCM group compared with its concentration in the other group (Figure 8B, right). An analysis of individual BAs confirmed that the CA, DCA, and taurodeoxycholic acid (TDCA) concentrations were increased in the iHFC + P and iHFC + MTZ groups, whereas the lithocholic acid (LCA) and MCA concentrations were reduced in these groups compared with their levels in the ND group (Figure 8C,D). Interestingly, the iHFC-mediated elevation of the CA concentration was enhanced by VCM; whereas DCA was almost undetectable in the VCM-treated mice (Figure 8C); and TDCA, a taurine-conjugated DCA, was also undetectable (Figure 8D). Notably, the concentrations of secondary 12αOH BAs, including LCA and MCA, in the iHFC + VCM group were severely reduced compared with their concentrations in the other groups (Figure 8C).

Because BAs exert their effects through FXR and TGR5 [4], we hypothesized that the changes in BA composition induced by the iHFC diet and antibiotic modification of gut microbiota may affect these receptors in the liver. We found that feeding mice the iHFC diet had no effects on the mRNA levels of these receptors (Figure S7). Interestingly, the expression of *TGR5* mRNA was markedly increased in the liver of mice in the iHFC + VCM group compared with its expression in the liver of mice in the iHFC + P and iHFC + MTZ groups after 8 weeks of treatment (Figure S7, right). *Enterobacteria*, especially Gram-positive bacteria, including *Clostridium*, *Lactobacillus*, and *Bifidobacterium*, have bile salt hydrolase (BSH), which deconjugates BAs by removing amino acids from conjugated BAs [31]. Deconjugated free BAs are dehydroxylated mainly in the large intestine. DCA and LCA are produced by 7α-dehydroxylation of CA and chenodeoxycholic acid (CDCA) by the intestinal bacteria, *Clostridium* XI and *Clostridium* XIVa, that express the BA inducible (bai) gene, such as *baiE* [13]. On the basis of the 16S rRNA sequencing analysis results, we performed a predictive functional profile analysis using the PICRUSt2 software Version 2021.2 to predict the metagenome expression of *BSH* and bai genes. In the iHFC + P group, the metagenome expression of *BSH* and bai genes, including *baiCD*, *baiH*, and *baiI*, was significantly reduced compared with their expression in the ND group (Figure S8). MTZ treatment showed similar results to placebo treatment (Figure S8). Notably, the metagenome expressions of *BSH* and various bai genes were almost undetectable in mice in the iHFC + VCM group (Figure S8). Thus, the iHFC diet and VCM treatment affected the level and balance of BA composition. Moreover, these data indicate that VCM treatment decreased the expression levels of BA-modifying bacterial genes, including *BSH* and other major bacterial BA dehydroxylases, thereby dramatically reducing the fecal levels of DCA, LCA, and TDCA and elevating the fecal levels of CA and TCA.

## 3. Discussion

In the present study, we dissected the links between gut microbiota, liver inflammation and advanced fibrosis using a newly established mouse NASH model with two antibiotics, MTZ and VCM. We showed that VCM, which targets Gram-positive organisms, exacerbated the progression of liver damage, steatohepatitis, and fibrosis in the iHFC-fed TSNO mice. VCM-treated mice had abundant F4/80^+^-recruited macrophages in their livers. Among these macrophages, the infiltration of CD11c^+^ subsets into the liver, forming hCLSs, was enhanced by VCM treatment. The co-localization of this macrophage subset with collagen in the liver of mice was greatly augmented in mice in the iHFC + VCM group. MTZ, which targets anaerobic organisms, had almost no effect on these changes. Additionally, the level and balance of BA composition were dramatically modulated by the VCM treatment, which decreased the expression of *BSH* and other major bacterial BA dehydroxylases, that were produced mainly by Gram-positive bacteria. Thus, we report that changes in inflammation and fibrosis in the liver by the iHFC diet can be modified by antibiotic-induced changes in gut microbiota. Their specific roles require further investigation, but the present findings provide novel insight into the importance of the gut microbe in the development of inflammation and advanced fibrosis in NASH by modulating BA metabolism.

In obese patients with type 2 diabetes mellitus, the intestinal barrier function is impaired, resulting in a leaky gut that facilitates LPS influx [17]. The role of LPS in the development of NAFLD and NASH has attracted some attention [32,33]. Circulating LPS levels and small intestinal permeability are increased in patients with NALFD, and these factors are associated with the severity of hepatic steatosis [32,34,35]. *Akkermansia muciniphila* is thought to contribute to improved intestinal barrier function [36]. We found that VCM treatment killed most Gram-positive bacteria (Figure S3), including *Ruminococcaceae* (*Clostridium* IV) and *Lachnospiraceae* (*Clostridium* XIVa), which are useful for the suppression of NASH development and produce short-chain fatty acids that are important for the gut environment and immunity. Additionally, most of the bacteria that survived the VCM treatment were Gram-negative bacteria that had LPS, which may be linked to liver inflammation. Furthermore, *Akkermansia muciniphila* was decreased in mice in the iHFC + P, iHFC + MTZ, and iHFC + VCM groups compared with its presence in the ND group (Figure S3), suggesting that leaky gut may be involved in the development of NASH in iHFC-fed mice, and that this is exacerbation by VCM treatment. Furthermore, *Alistipes*, which is inversely related to fibrosis, was decreased in the iHFC + P and iHFC + VCM groups compared with its presence in the ND group (Figure S3). These mechanisms may be involved in the regulation of intestinal bacteria and the development of NASH triggered by the iHFC diet and VCM treatment. Further studies are required to clarify the details of these mechanisms.

VCM treatment dramatically modulated the level and balance of BA composition in the iHFC-fed mice (Figure 8) by decreasing the expression of *BSH* and other major bacterial BA dehydroxylases (Figure S8) that are mainly produced by Gram-positive bacteria, such as *Lactobacillus*. The level and balance of BA composition have a crucial role in intestinal barrier function [9,10]; in particular, secondary BAs are key factors responsible for leaky gut [14]. Previous studies showed that the level of primary 12αOH BAs in the enterohepatic circulation was selectively increased by feeding an HFD [11,12], and the level of primary BAs in the small intestine has been shown to be higher than that of secondary BAs in the large intestine [12]. In rats on a CA-supplemented diet, VCM treatment increased the TCA concentration in the ileum [37], whereas DCA was almost undetectable at the same site, and fecal CA concentration was high in rats fed the CA with VCM treatment [37]. These data are similar to our present findings (Figure 8) and confirm that VCM treatment suppressed the 7α-dehydroxylation of CA in the large intestine. Moreover, TCA selectively affects the gut permeability in the ileum, which is the site for reabsorbing conjugated BAs [37]. In addition, the effect of the TCA on ileal permeability is much stronger than that of DCA in the colon for gut leakiness [37]. Therefore, we suggest that the iHFC diet with VCM treatment may disrupt gut permeability by increasing TCA levels in the small intestine and induce liver inflammation by enterohepatic circulation in our mouse NASH model. Future studies are needed to investigate the TCA concentrations and mucosal barrier function in the small intestine using the mouse NASH model.

There are two major receptors of BAs: FXR and TGR5 [4]. FXR is known to regulate BA and lipid metabolism [38,39]. TGR5 is located on immune cells, such as macrophages [40,41], and has anti-inflammatory effects [42]. Secondary BAs such as DCA and TDCA have high affinities for TGR5 [43]. DCA and LCA were shown to reduce NAFLD by activating FXR and TGR5 in the intestinal tract and inducing the production of FGF19/21 and GLP-1, respectively [5,44]. We found that secondary BAs such as DCA and LCA were markedly decreased by VCM, which suggested that the activation of FXR and TGR5 by these BAs may have been suppressed by the VCM treatment. This could explain the exacerbation of NASH development with VCM treatment. We also found that VCM treatment markedly increased *TGR5* mRNA expression in the liver of the iHFC-fed mice (Figure S7, right). TGR5 is highly expressed in macrophages and has an important role in regulating inflammation [40,41]. Because VCM treatment enhanced iHFC diet-induced macrophage infiltration in the liver (Figure 4D,E and Figure 5E), this macrophage infiltration probably reflects the elevated *TGR5* mRNA expression.

**Figure 9:**
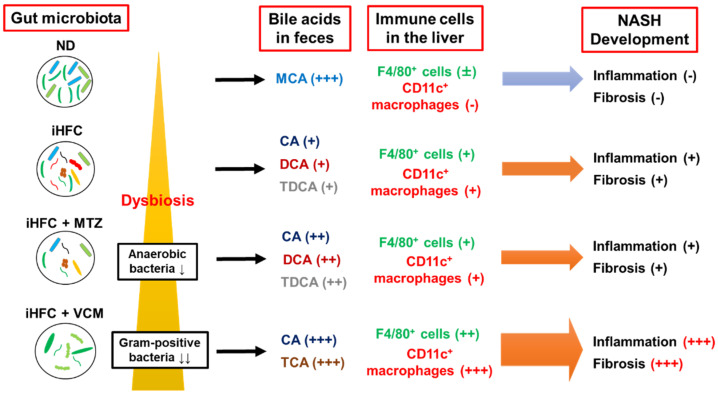
A summary of the effects of antibiotics treatment on BA metabolism, dynamics of immune cells, and development of inflammation and fibrosis in the iHFC diet-induced mouse NASH model.

In summary, VCM treatment had profound effects on the gut microbiota that resulted in changes in BA metabolism and the development of liver inflammation and advanced fibrosis in a novel diet-induced mouse NASH model (Figure 9). The present findings may provide information that will aid in the development of therapeutic agents for NASH that target the gut microbiome. On the other hand, this study has several limitations (e.g., the lack of comparison with relevant data sets of mice fed ND+P, ND+MTZ, and ND+VCM; the lack of information on inflammation-related gene expression in the gut; the low numerosity of the sample size). Future investigations will clarify these issues as well as the details of Gram-positive bacteria and BAs involved in the development of liver inflammation and fibrosis.

## 4. Materials and Methods

### 4.1. Mice Used in This Study

Male TSNO mice (6-weeks-old) were purchased from the Institute of Animal Reproduction (Ibaraki, Japan) and maintained in microisolator cages under specific pathogen-free conditions in the animal facility of Toyama Prefectural University under standard light conditions (12 h light/dark cycle) and with free access to water and food. Seven-week-old male TSNO mice were fed ad libitum either a normal diet (ND) (MF, Oriental-Yeast, Tokyo, Japan) or an iHFC diet, that was high in fat, cholesterol, and cholate (69.5% standard chow, 28.75% palm oil, 1.25% cholesterol, and 0.5% cholate) (Hayashi Kasei, Osaka, Japan) for the indicated periods. For the antibiotic treatment, 7-week-old mice were fed the ND or iHFC diet for 4 or 8 weeks, and simultaneously treated with either placebo, VCM (Nacalai Tesque, Kyoto, Japan) (50 µg/body weight), or MTZ (Nacalai Tesque) (50 µg/body weight) by oral gavage for 5 days a week. These antibiotics were diluted with sterilized water and prepared in total to be 200 µL/mouse. The animal care policies and procedures/protocol used in the experiments were approved by the Animal Experiment Ethics Committee of Toyama Prefectural University (Approval No. R1-2 and R3-6).

### 4.2. Plasma Biochemical Analysis

Blood samples were collected from the inferior vena cava, and plasma samples were also collected. Plasma levels of alanine aminotransferase (ALT), total cholesterol (T-CHO), and triglyceride (TG) were measured by DRI-CHEM NX700 (Fujifilm, Tokyo, Japan) following the manufacturer’s instruction.

### 4.3. Isolation of Non-Parenchymal Cells from Liver

To isolate non-parenchymal cells from the liver, mice were anesthetized (isoflurane) and perfusion was performed with phosphate-buffered saline. The isolation of non-parenchymal cells was performed using a Liver Dissociation Kit (Miltenyi Biotech, Bergisch Gladbach, Germany) following the manufacturer’s instructions. The cell suspension was passed through a cell strainer (100 µm) and used for the flow cytometry analysis.

### 4.4. Flow Cytometry Analysis

The non-parenchymal cells (2 × 10^5^) were incubated with anti-mouse FcγR (2.4G2) to block binding of the fluorescence-labeled antibodies to FcγR. After 20 min, the cells were stained with predetermined optimal concentrations of the respective antibodies. Then, 7-amino-actinomycin D (7-AAD) (BD Biosciences, San Diego, CA, USA) was used to exclude dead cells. Flow cytometry analyses were conducted on a FACSCantoII (Becton Dickinson & Co., Mountain View, CA, USA), and the data were analyzed with Flowjo software Version 10.8.1 (BD Biosciences). The antibodies for flow cytometry are listed in Table S1.

### 4.5. Preparation of RNA and cDNA

Total RNA was extracted using a NucleoSpin RNA Mini Kit (Macherey-Nagel, Düren, Germany) following the manufacturer’s instructions. RNA was reverse transcribed using a PrimeScript^®^ RT Reagent Kit (Takara Bio Inc., Shiga, Japan) following the manufacturer’s instructions.

### 4.6. Quantitative Real-Time PCR

A qRT-PCR was performed with a FastStart Universal Probe Master (Roche Applied Science, Mannheim, Germany) and analyzed with a CFX96 Touch™ Real-Time PCR Detection System (Bio-Rad, Hercules, CA, USA) following the manufacturers’ instructions. Relative transcript abundance was normalized to that of Hprt mRNA. The information for the TaqMan primer/probes (Applied Biosystems, Carlsbad, CA, USA) used for the qRT-PCR is listed in Table S2.

### 4.7. Histological and Immunohistochemistry Analysis

Portions of the liver were excised and fixed immediately with 4% formaldehyde at room temperature. Paraffin-embedded tissue sections were cut into 4 μm slices and placed on slides. Sections were stained with hematoxylin and eosin or Sirius red, according to standard procedures. Anti-F4/80, anti-CD11c, and anti-Ly6C antibodies were purchased from Cedarlane Laboratories (Burlington, Ontario, Canada), Invitrogen (Waltham, MA, USA), and Abcam (Cambridge, MA, USA), respectively. Positive areas for F4/80 and Sirius red were measured using ImageJ software Version 1.53t [45]. Histologic steatosis, lobular inflammation, and hepatocyte ballooning were assessed according to the criteria proposed by Kleiner et al. [46]. All histological analyses were performed by pathologists (K.Tsuneyama and M.I-S.), and the histological scores and grade were determined in a blinded manner.

### 4.8. Fluorescent Immunohistochemistry Analysis

We incubated 7 μm frozen sections with anti-CD11c (Invitrogen) and secondary antibody (anti-hamster IgG, Southern Biotech, Birmingham, AL, USA), and sequentially incubated using a TSA Fluorescein System (Akoya Biosciences, Marlborough, MA, USA). The sections were also incubated with anti-collagen type 1 (Novotec, Bron, France) and secondary antibody (anti-rabbit IgG Alexa Fluor 594, Abcam). Finally, the sections were incubated with DAPI (Invitrogen). Images were acquired using a BX50 microscope and its imaging system (Olympus, Tokyo, Japan). The positive signal of each co-localized area was selected according to the method of Tolivia et al. (Figure S6) [47] and analyzed using Adobe Photoshop CS software, version 8.

### 4.9. Metagenomic 16S rRNA Sequencing

One fresh fecal pellet was collected from each mouse and stored at −80 °C. For DNA extraction, the frozen stool samples were transferred to a lysis buffer from a ZymoBIOMICS DNA Miniprep Kit (Zymo Research, Irvine, CA, USA), homogenized using a Disruptor Genie (Scientific Industries, Bohemia, NY, USA; 3000 rpm for 20 min), and extracted using a ZymoBIOMICS DNA Miniprep Kit, according to the manufacturer’s instructions.

For sequencing, 16S rRNA gene sequence libraries were prepared according to the Illumina (San Diego, CA, USA) protocol, as described previously [48]. The final libraries were pooled, diluted to 4 nM in 5 mM Tris-HCl buffer, and sequenced using a Miseq System with a 600-Cycle Kit (Illumina).

A prediction of functional profiles from the 16S rRNA data sets was conducted using the Phylogenetic Investigation of Communities by Reconstruction of Unobserved States (PICRUSt2) software and the Kyoto Encyclopedia of Genes and Genomes (KEGG) database release 70.0. Pathways involved in human diseases were removed because they apply to human cells or tissues.

### 4.10. Bacterial Community Analysis

The Quantitative Insight Into Microbial Ecology 1 (QIIME1) pipeline, an open-source bioinformatics tool for the analysis of raw microbiome DNA sequencing data, was used to investigate the bacterial composition as described previously [48]. The taxonomy assignment was performed using the EzbioCloud reference database.

### 4.11. Bile Acid Analysis

Fecal BAs were extracted by homogenizing dried feces (30–60 mg) in a mixture of 0.5 mL methanol, 0.8 mL acetonitrile, and 0.2 mL 28% (*w*/*v*) ammonium hydroxide with 100 nM 23-dinor-deoxycholic acid as an internal standard (Steraloids, Newport, RI, USA). The fecal homogenates were centrifuged, and the resultant supernatants were applied to solid-phase extraction columns to obtain fractions containing BAs as described previously [49]. The BA levels in the fecal samples were determined by liquid chromatography-electrospray ionization-mass spectrometry (LC-ESI-MS) as described previously [49].

### 4.12. Statistical Analysis

Statistical significance was evaluated by two-way ANOVA followed by the post hoc Tukey test for multiple comparisons. A statistical analysis was performed using GraphPad Prism 9 software (GraphPad; San Diego, CA, USA). When we analyzed the parametric tests, we first performed an F test on Prism 9 software to confirm that the *p*-value of the F test was >0.05 and equally distributed. *p* < 0.05 was considered statistically significant. The results are presented as the mean ± standard deviation (SD).

## Data Availability

The data that support the findings of this study are available from the corresponding author upon reasonable request.

## Supplementary Materials

The following supporting information can be downloaded at: https://www.mdpi.com/article/10.3390/ijms24044050/s1.

## Abbreviations

ALT	alanine aminotransferase
aSMA	Alpha-smooth muscle actin
BA	bile acid
bai	BA inducible
BSH	bile salt hydrolase
CA	cholic acid
CCl4	carbon tetrachloride
CDCA	chenodeoxycholic acid
DCA	deoxycholic acid
FXR	farnesoid X receptor
HCC	hepatocellular carcinoma
hCLS	hepatic crown-like structure
HDCA	hyodeoxycholic acid
HFD	high-fat diet
KC	kupffer cell
LCA	lithocholic acid
LPS	lipopolysaccharide
MCA	muricholic acid
MTZ	metronidazole
NAFL	non-alcoholic fatty liver
NAFLD	non-alcoholic fatty liver disease
NASH	non-alcoholic steatohepatitis
ND	normal diet
TCA	taurocholic acid
TCDCA	taurochenodeoxycholic acid
T-CHO	total cholesterol
TDCA	taurodeoxycholic acid
TG	triglyceride
TGR5	Takeda G protein-coupled receptor 5
THDCA	taurohyodeoxycholic acid
TMCA	tauromuricholic acid
TUDCA	tauroursodeoxycholic acid
TSNO	Tsumura-Suzuki non-obese
UDCA	ursodeoxycholic acid
VCM	vancomycin
12aOH	12a-hydroxylated
