# Supplementary material for: Impact of Vancomycin Treatment and Gut Microbiota on Bile Acid Metabolism and the Development of Non-Alcoholic Steatohepatitis in Mice
Source: Int J Mol Sci. 2023 Feb 17;24(4):4050. doi: 10.3390/ijms24044050 (PMC9967260; doi:10.3390/ijms24044050)
Supplement: Supplementary file 1 [file ijms-24-04050-s001.zip › ijms-2160374-supplementary.pdf]

Supplementary Table S1. Antibodies for flow cytometry.

The antibodies for flow cytometry are listed. The antibodies were purchased from BD Pharmingen (San Diego, CA) or BioLegend (San Diego, CA).

Antibody	Clone	Conjugate	Source
Anti-CD11c	HL3	PE	BD Pharmingen
Anti-CD11b/Mac-1	M1/70	APC-Cy7	BD Pharmingen
Anti-CD45	30-F11	APC	BioLegend
Anti-F4/80	BM8	PE	BioLegend
Anti-F4/80	BM8	FITC	BioLegend
Anti-Ly6C	HK1.4	FITC	BioLegend

Supplementary Table S2. Primers for RT-qPCR.

The primers were purchased from Applied Biosystems (Waltham, MA).

Gene	Gene Symbol	Gene Name	Assay ID
Hprt	Hprt1	hypoxanthine guanine phosphoribosyl transferase	Mm00446968_m1
TNF-a	Tnf	tumor necrosis factor	Mm00443258_m1
Il-1b	Il1b	interleukin 1 beta	Mm01336189_m1
Il-6	Il6	interleukin 6	Mm00446191_m1
MCP-1	Ccl2	chemokine (C-C motif) ligand 2	Mm00441243_g1
iNOS	Nos2	nitric oxide synthase 2, inducible	Mm01309898_m1
MPO	Mpo	myeloperoxidase	Mm01298424_m1
CCR2	Ccr2	chemokine (C-C motif) receptor 2	Mm99999051_gH
F4/80	Adgre1	adhesion G protein-coupled receptor E1	Mm00802530_m1
CD11c	Itgax	integrin alpha X	Mm00498698_m1
Arg-1	Arg1	arginase 1	Mm01190441_g1
MMP-2	Mmp2	matrix metallopeptidase 2	Mm00439498_m1
Tgfb-1	Tgfb1	transforming growth factor, beta 1	Mm01178820_m1
TIMP-1	Timp1	tissue inhibitor of metalloproteinase 1	Mm00441818_m1
Col1a-1	Col1a1	collagen, type 1, alpha 1	Mm00801666_g1
a-SMA	Acta2	actin, alpha 2, smooth muscle, aorta	Mm00725412_s1
Desmin	Des	desmin	Mm00802455_m1
Fxr	Nr1h4	nuclear receptor subfamily 1, group H, member 4	Mm00436425_m1
TGR5	Gpbar1	G protein-coupled bile acid receptor 1	Mm04212121_s1

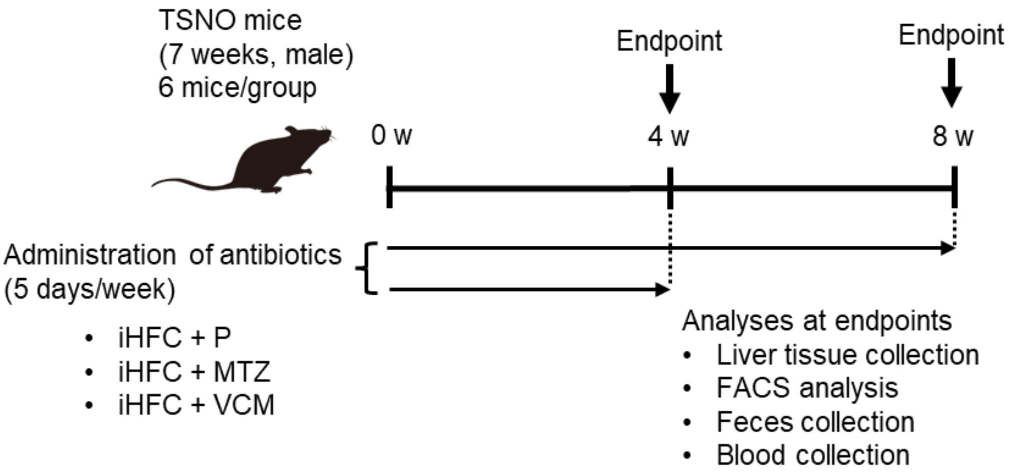

Supplementary Figure S1. Schematic overview of the study design for antibiotic modification of the gut microbiota using TSNO mice on the iHFC diet.

Seven-week-old mice were fed with the iHFC diet for 4 or 8 weeks, and simultaneously treated with either placebo (sterilized water) (iHFC + P), metronidazole (iHFC + MTZ) (50 µg/body weight), or vancomycin (iHFC + VCM) (50 µg/body weight) by oral gavage for 5 days a week (6 mice per group). After euthanasia, mice were analyzed as depicted.

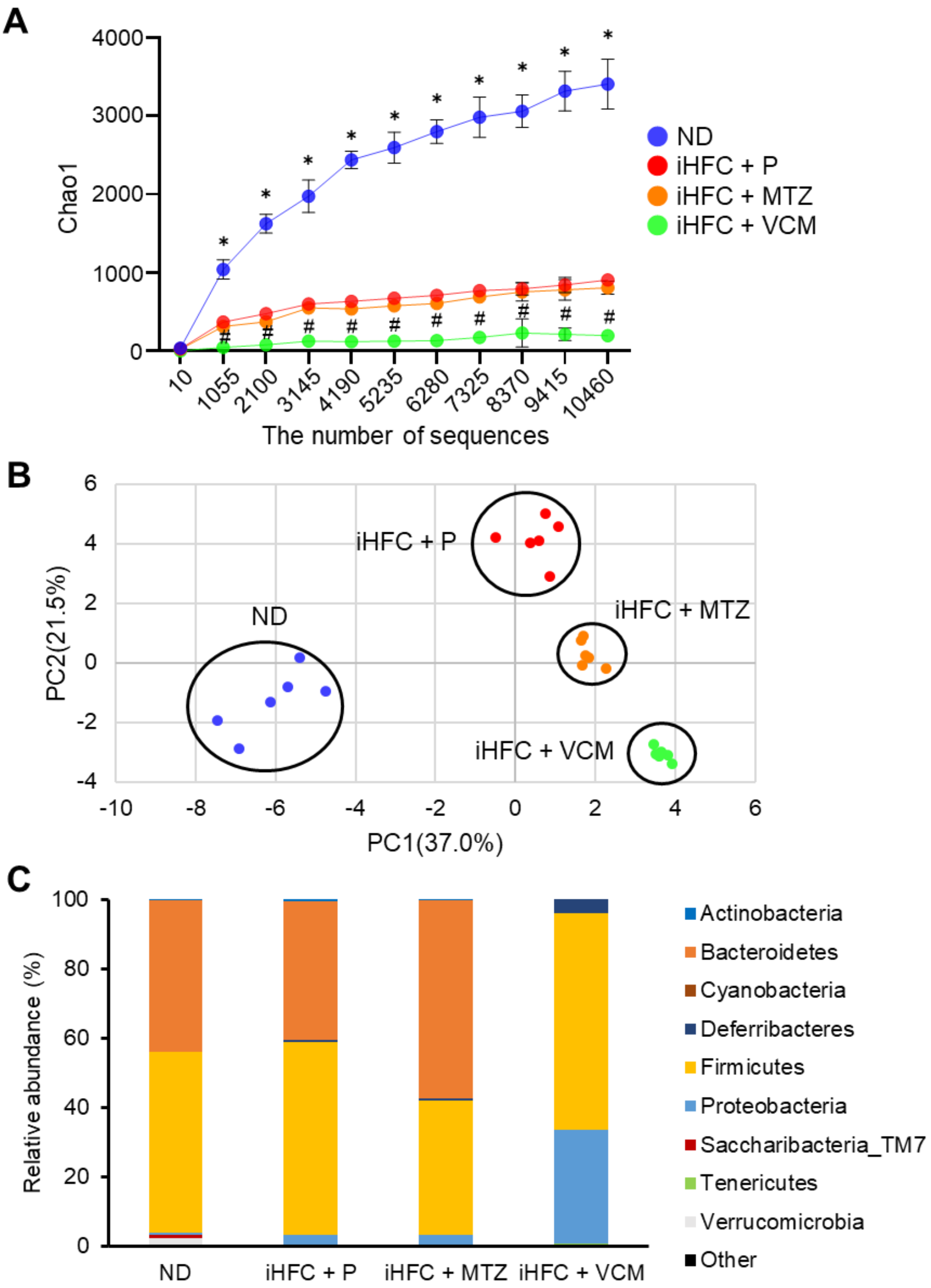

Supplementary Figure S2. Effect of antibiotic treatment on the gut microbiome in iHFC-fed TSNO mice.

Fecal samples were collected from ND- or iHFC diet-fed TSNO mice after 4 weeks of placebo or antibiotics treatment. (A) Effect of diet and antibiotics treatment on the α-diversity (Chao1) of gut bacteria. $^{*}p < 0.001$ for ND vs iHFC + P, $^{\#}p < 0.001$ for iHFC + P vs iHFC + VCM. Statistical significance was evaluated by 2-way ANOVA followed by post-hoc Tukey test. (B) Principal component analysis of β-diversity values. (C) Representation of relative abundance of bacterial phyla in the fecal microbiota of mice from each group (n = 6 per group).

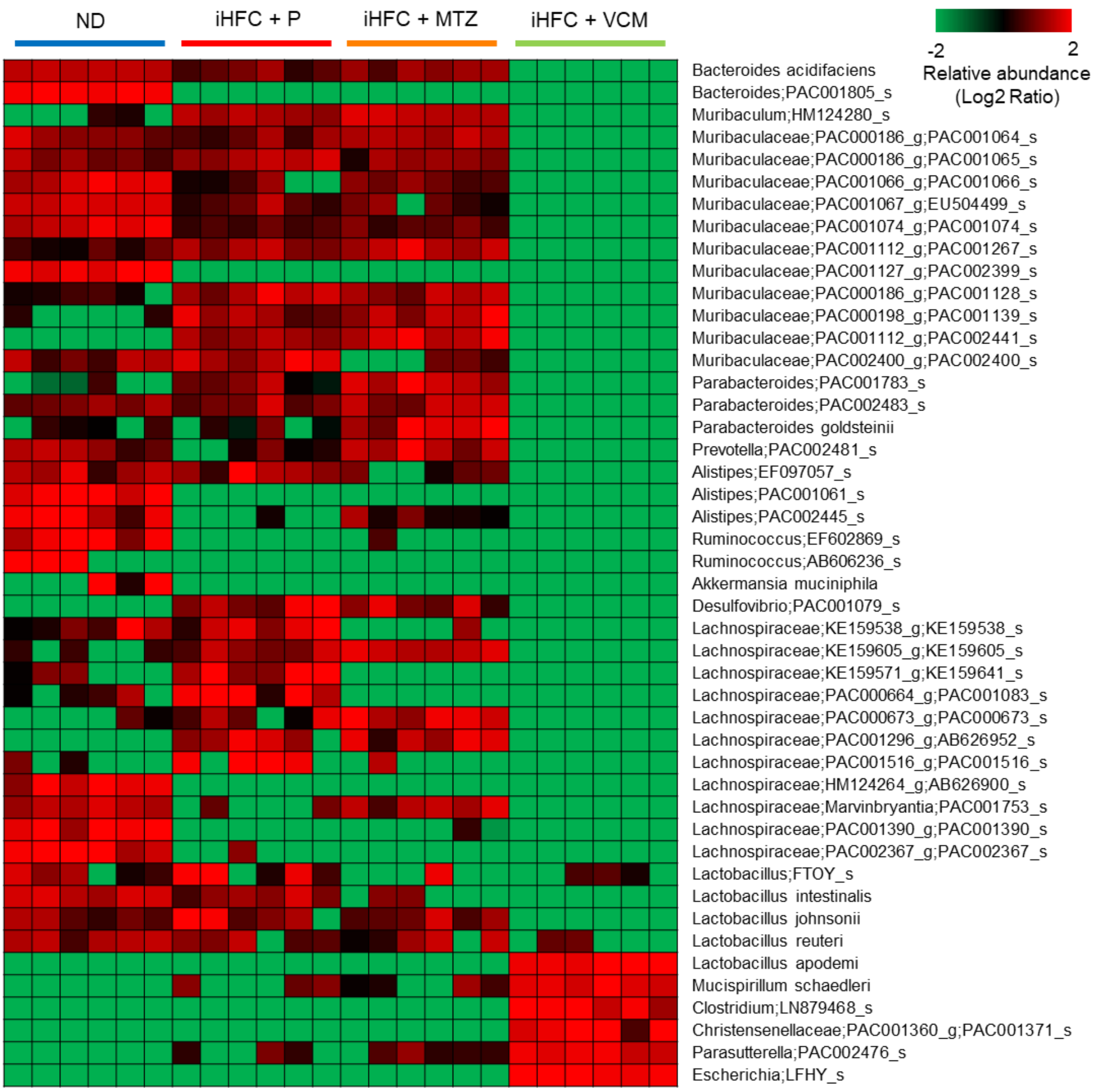

Supplementary Figure S3. Effect of antibiotic treatment on the gut microbiome in iHFC-fed TSNO mice. Related to Supplementary Figure S2.

Effect of diet and antibiotic treatment on the abundance of shared bacteria taxa (n = 6 per group). A mean abundance of more than 1% was extracted.

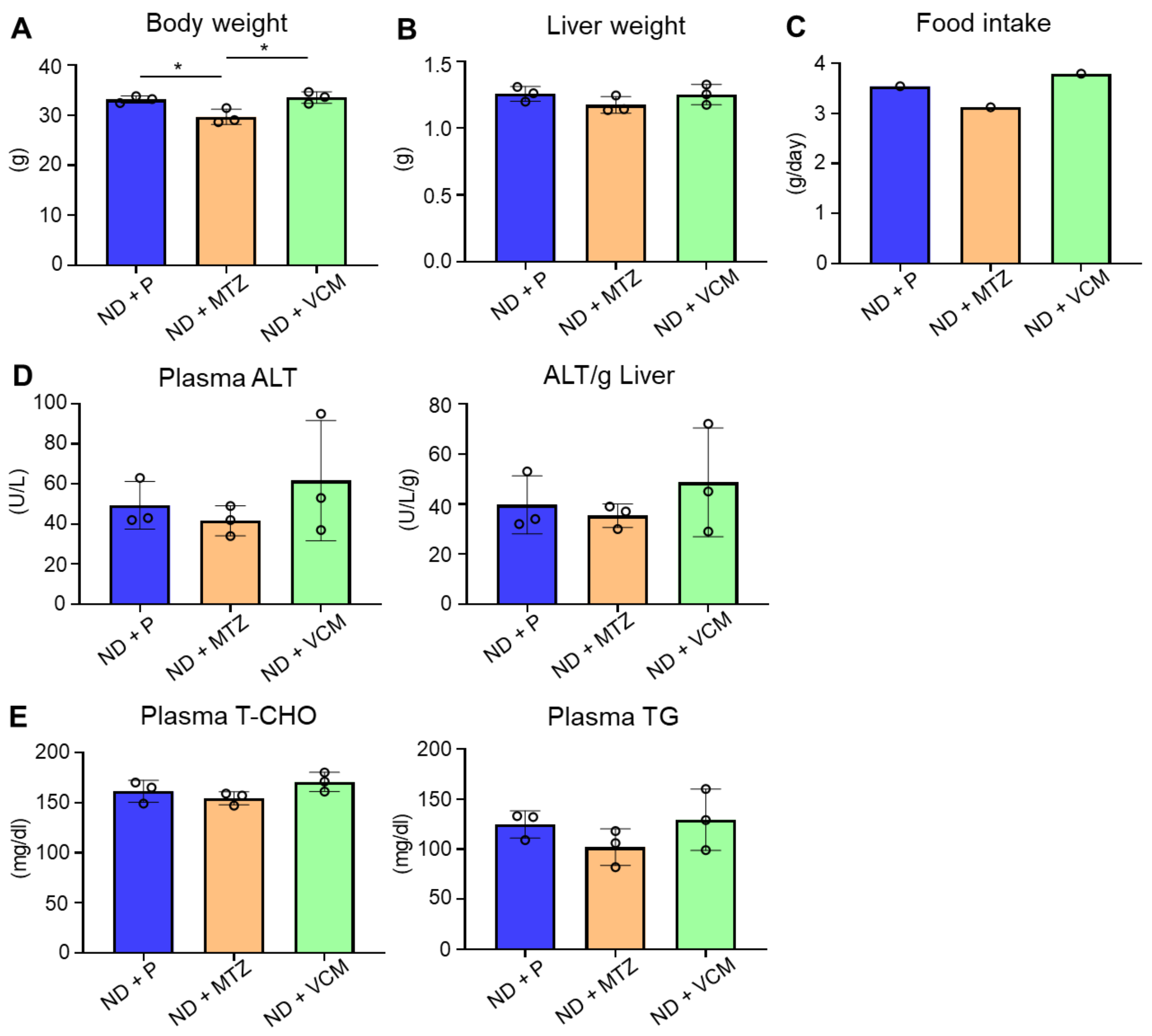

Supplementary Figure S4. Antibiotic treatment does not affect body weight, liver weight, food intake, and plasma ALT, T-CHO, and TG levels of TSNO mice on the ND diet. Seven-week-old male TSNO mice were fed with the ND for 4 weeks and simultaneously treated with either metronidazole (ND + MTZ) (50 µg/body weight) or vancomycin (ND + VCM) (50 µg/body weight) by oral gavage for 5 days a week (n = 3 per group). (A-C) Body weights, liver weights, and daily food intakes were measured. (D) Plasma ALT levels were measured, and plasma ALT/g liver levels were also calculated. (E) Plasma T-CHO and TG levels were measured. Data are shown as means ± SD. *$p < 0.05$. Statistical significance was evaluated by 2-way ANOVA followed by post-hoc Tukey test.

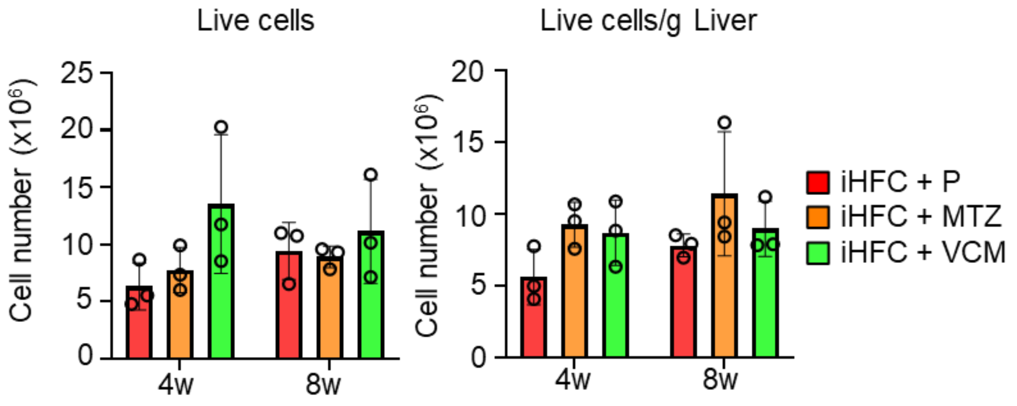

Supplementary Figure S5. Flow cytometry analysis of non-parenchymal cells in the liver from placebo- or antibiotics-treated TSNO mice.

Left, cell number of live non-parenchymal cells of the livers from placebo- or antibiotics-treated TSNO mice fed the iHFC diet for the indicated time periods (n = 3 per group). Right, cell number of live non-parenchymal cells per liver weight (g) were also calculated (n = 3 per group). Data are shown as means ± SD.

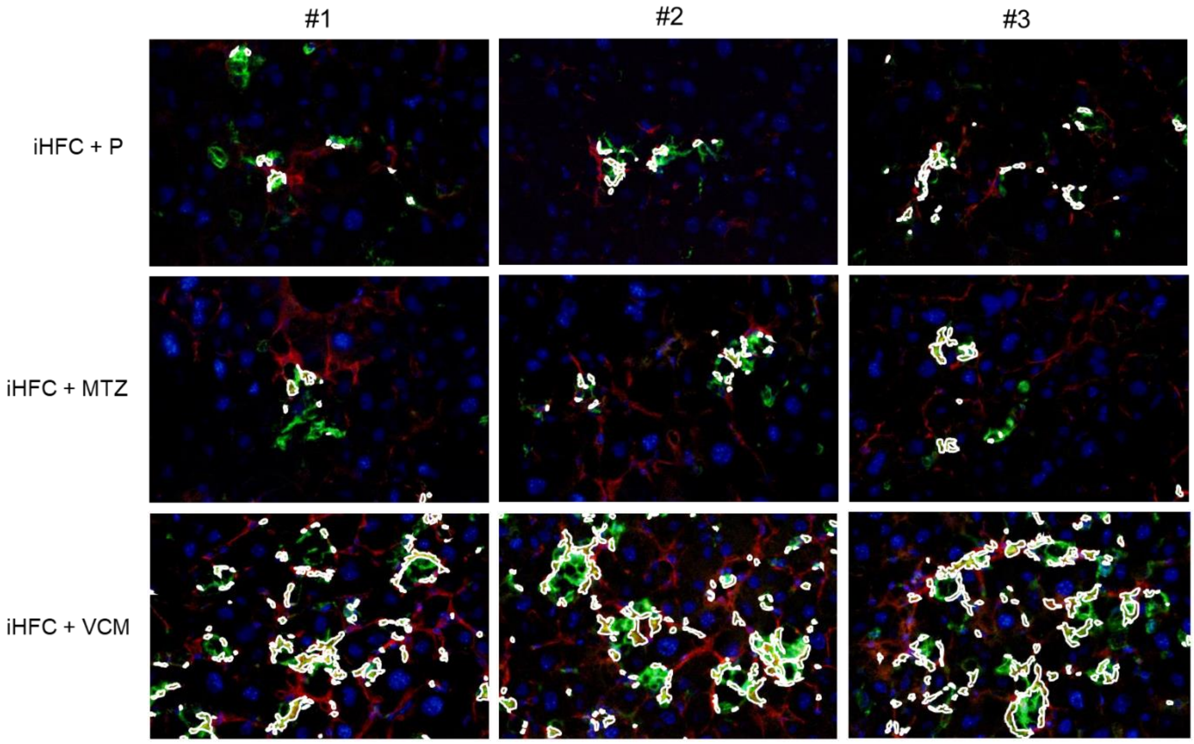

Supplementary Figure S6. Colocalized areas of CD11c and collagen immunostainings in the liver from TSNO mice.

Representative histological images (40x magnification) of fluorescent immunohisto-chemistry for CD11c, collagen type 1, and DAPI of the livers from placebo or antibiotics-treated TSNO mice on the iHFC diet for 8 weeks (3 mice/group). The positive signal of each colocalized area was selected according to the method of Tolivia J et al. (ref. 47).

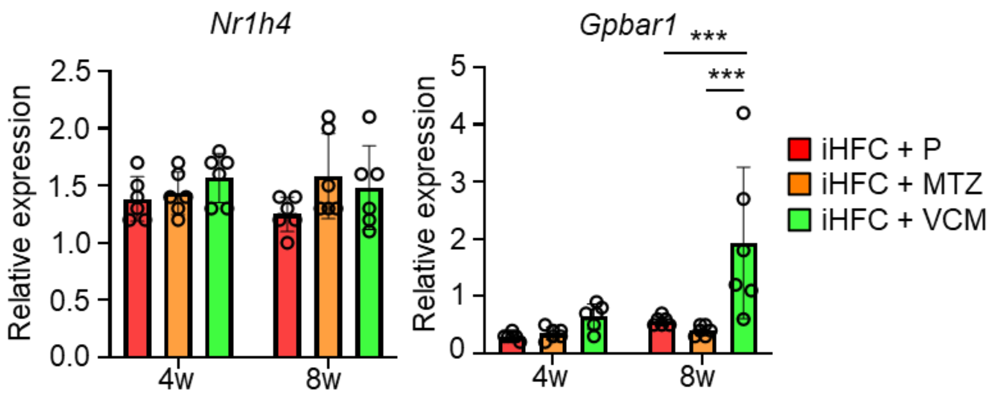

Supplementary Figure S7. VCM treatment increases the levels of TGR5 gene expression in the liver of iHFC diet-fed TSNO mice.

RT-qPCR of FXR (*Nr1h4)* and TGR5 (*Gpbar1*) mRNA in the livers from placebo- or antibiotics-treated TSNO mice fed the iHFC diet for the indicated time periods (n = 6 per group). Data are shown as means ± SD. ****p* < 0.001. Statistical significance was evaluated by 2-way ANOVA followed by post-hoc Tukey test.

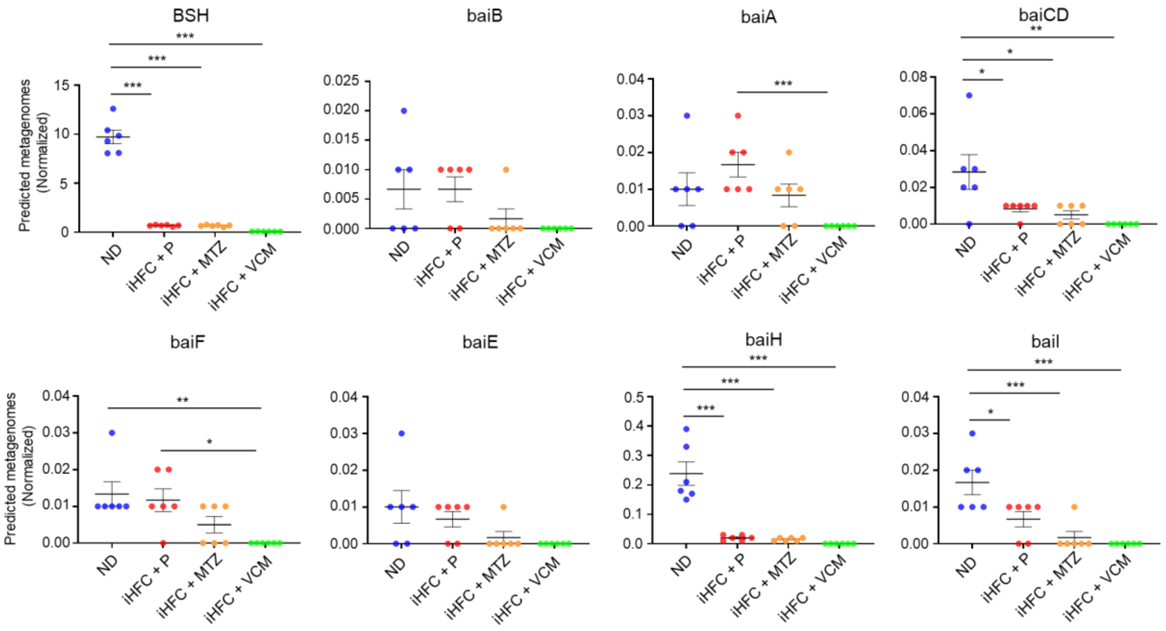

Supplementary Figure S8. iHFC diet feeding and antibiotic treatment on the iHFC diet markedly reduce the expression levels of bile salt hydrolase (BSH) and various bile-acid 7α-hydroxylase metagenomes.

A predictive functional profile analysis (PICRUSt2 analysis) was performed to predict the metagenome expression of BSH and bai genes based on the results of 16S rRNA sequencing analysis (Related to Supplementary Figure 2 and 3). Fecal samples were collected from ND- or iHFC diet-fed TSNO mice after 4 weeks of placebo or antibiotics treatment (n = 6 per group). Data are shown as means ± SD. **p* < 0.05, ***p* < 0.01, ****p* < 0.001. Statistical significance was evaluated by 2-way ANOVA followed by post-hoc Tukey test.
